# ACC.20: Impact of social media at the virtual scientific sessions during the COVID‐19 pandemic

**DOI:** 10.1002/clc.23387

**Published:** 2020-07-03

**Authors:** Graham Mackenzie, Martha Gulati

**Affiliations:** ^1^ Department of Public Health NHS Education for Scotland Scotland UK; ^2^ Division of Cardiology University of Arizona Phoenix Arizona USA

**Keywords:** Covid‐19, ACC20, impact of social media, Virtual ACC Scientific Sessions

## Abstract

**Background:**

The COVID‐19 pandemic led to the American College of Cardiology (ACC) Annual Scientific Session 2020 (ACC.20) being held as a virtual event.

**Hypothesis:**

Social media activity around a virtual event might be quite different to that of a physical meeting. The goal of this study was to assess impact of ACC.20 through Twitter and compare it to ACC.19.

**Methods:**

Data were extracted using NodeXL, with analysis in Excel.

**Results:**

ACC.20‐related tweeting was demonstrated globally. However tweeting and participants fell substantially for ACC.20. Tweeting, participation and tweet views were overestimated by the most widely used social media analysis tool used at medical conferences (Symplur).

**Conclusion:**

Comparing the 2019 and 2020 Scientific Sessions, the global cardiology community continued to communicate despite COVID‐19, but with reduced social media activity potentially due to the briefer format, no physical interaction and private virtual chatroom during live sessions, reducing visibility of new cardiology research findings.

## 1 INTRODUCTION

The COVID‐19 pandemic has meant that healthcare workers and organizations have had to make unprecedented changes, both in work, education and networking. Individual healthcare workers and units have had to resort to virtual learning, often focussed specifically on COVID‐19. Many international conferences scheduled for 2020 have been canceled outright. The American College of Cardiology (ACC) made the decision to move its annual Scientific Session into a virtual event (ACC.20), scheduled for the same 3 days in March 2020 (Saturday 28 March‐Monday 31 March) with limited programming. The event was hosted jointly with the World Heart Federation's World Congress of Cardiology (WCC).

Social media has become an important part of medical conferences. It provides opportunities for wider dissemination of key findings from the conference, feedback from peers across the world and networking. Analysis of the 2019 ACC Scientific Sessions (ACC.19) described social media interactions and content in detail, identifying the challenges of separating out cardiology tweets from those posted about other events with the same ACC acronym.^1^ Social media activity around a virtual event might be expected to be quite different to that around a physical meeting. The analysis presented here looks at social media activity related to ACC.20/WCC and compares this with ACC.19.

## 2 METHODS

Data were extracted using NodeXL,^2^ with analysis in Excel, as described in the ACC.19 paper.^1^ The hashtags used were the official hashtags for the virtual event (#ACC20, #WCCardio), and some tweeters used #ACC2020. A further 77 tweets were identified using the term “ACC.20” without hashtags, but these were not included in this analysis as the comparable data were not available for ACC.19.

ACC.19 data were re‐analyzed to allow direct comparison with the ACC.20 data, focussing on the day before the events, the 3 days of the conference, and the day after the events (15‐19 March 2019 and 27‐31 March 2020, coordinated universal time). Data on individual retweets were used rather than the aggregate count of retweets recorded in tweets, to allow a direct comparison of activity over the 5‐day period rather than retweets accumulated after this period (see Supporting Information Data S1). Preliminary data and findings and other mapping data were shared in a tweet thread, including the global spread of ACC.20 related tweets and retweets.^3^ A summary of tweets posted during the 3 days of the virtual event was shared via a Wakelet summary.^4^

The most widely disseminated tweets were viewed and one tweet with a large number of interactions selected^5^ for further mapping to document the branching structure of replies and quoting tweets (collectively referred to as “responses” in this paper). These responses were identified by viewing and expanding replies in Twitter in an internet browser, recording the tweet URL and searching for quoting tweets by copying the tweet URL into the Twitter search box after removing the leading “https://www.”, repeating until no further responses were found.^6^ The 19 character tweet IDs for individual responses were then mapped using NodeXL, which also collects information about tweeters and numbers of retweets obtained for individual tweets.^7^

Finally, the number of tweets was recorded using the Symplur healthcare hashtags website, as this is a tool commonly used and shared in conference tweeting to track headline statistics for single hashtags.^8^ Symplur provides estimates of “tweets” (which they calculate by adding tweets and retweets), and “tweeters” (calculated by adding tweeters and retweeters) for single, registered hashtags. Symplur also provides estimates of “impressions” (number of times a tweet has been displayed on a Twitter‐enabled device) by adding the number of tweets and retweets made by a tweeter and multiplying by total number of followers for that tweeter. Looking at #ACC20 by itself, and focusing on the American College of Cardiology (@ACCinTouch) Twitter account, Symplur data were compared with NodeXL data (separate records of tweets, retweets, tweeters and retweeters) and Twitter Analytics (a record of impressions direct from Twitter),^9^ and the ACCinTouch Twitter feed (providing a record of retweets).^10^

## 3 RESULTS

**FIGURE 1:**
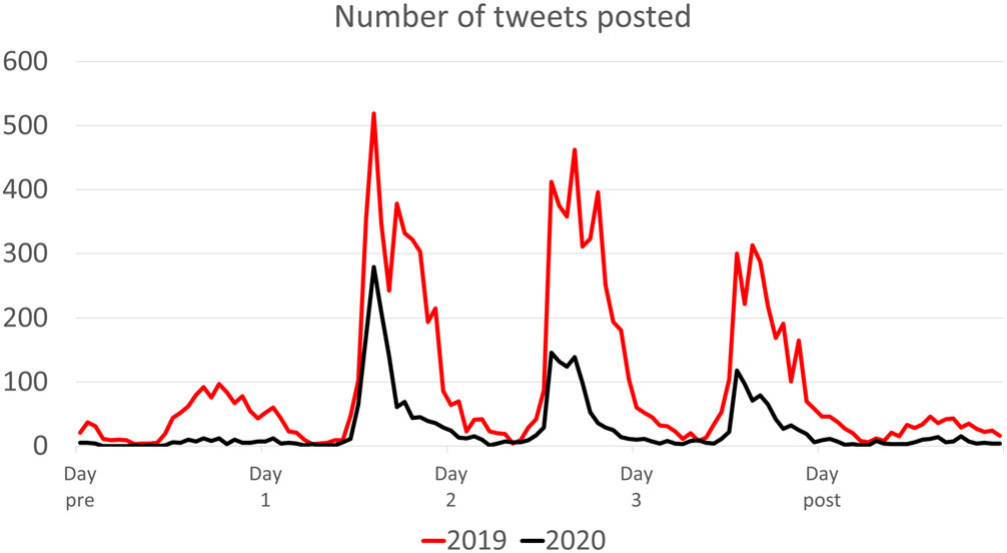
Number of tweets posted, comparing equivalent 5‐day period for ACC.19 and ACC.20 (Source: NodeXL)

Table 1 summarizes the figures for ACC.19 and ACC.20/WCC. The number of tweets, retweets, tweeters and retweeters all fell substantially for ACC.20/WCC. There was more “hashtag drift” during ACC.20/WCC than ACC.19, with 14.4% of tweets using just #ACC2020 compared with 4.3% using #ACC2019 the previous year. Overall, 107 ACC.20/WCC tweeters received 80% of retweets, representing just 13.8% of all tweeters; conversely, 256 (32.7%) tweeters received no retweets, suggesting minimal impact of their tweets. The duration of tweeting and retweeting each day was shorter for ACC.20/WCC (Figures 1 and S1).

**TABLE 1:** Tweets and retweets, tweeters and retweeters at ACC.19 and ACC.20

	ACC.19	ACC.20/WCC
Tweets posted	11 757	3147
% Tweets using official hashtag(s)	95.7% used #ACC19	84.9% used #ACC20 0.7% used just #WCCardio
Retweets made	29 929	8566
Number of tweeters	2039	777
Number of retweeters	7647	3272
Tweeters or retweeters	8540	3679

*Note:* Source: NodeXL extracts.

Table 2 summarizes the type of contribution made—whether tweeting and/or retweeting—for ACC.19 and ACC.20/WCC. For both years the majority contribution was “just retweeting.” For ACC.20/ WCC, 1219 tweeters and/or retweeters (33.1%) had also contributed to ACC.19. For these 1219 tweeters/retweeters, the nature of contribution was at the same level for 64.6% (eg, just retweeted for both years), while 28.5% reduced their level of contribution (eg, tweeted and retweeted in 2019, just retweeted in 2020), and 7.0% increased their contribution (eg, just tweeted in 2019, both tweeted and retweeted in 2020).

**TABLE 2:** Tweeting and/or retweeting patterns at ACC.19 and ACC.20/WCC

	ACC.19	ACC.20/WCC
Just tweeters	893 (10.5%)	407 (11.1%)
Just retweeters	6501 (76.1%)	2902 (78.9%)
Tweeters and retweeters	1146 (13.4%)	370 (10.1%)

*Note:* Source: NodeXL extracts.

Table 3 lists the top 20 tweeters for ACC.20/WCC based on the number of retweets received. Comparing the 2019 and 2020 lists, 6 of these top 20 tweeters were also in the top 20 list for ACC.19, and a further 8 were in the top 100 for ACC.19. Altogether, these 14 tweeters received 38% of retweets in 2020. Five of the other six tweeters in the 2020 top 20 did not tweet during ACC.19, while cardioinfo_it posted 6 tweets, receiving 5 retweets.

**TABLE 3:** Top 20 tweeters for ACC.20/ WCC by number of retweets received (shaded tweeters were in top 100 tweeters for ACC.19)

Tweeter	Retweets received	Tweets posted	% Of RTs received	Cumulative % of RTs received	Followers
accintouch	830	185	9.0	9.0	80 649
nejm	685	11	7.5	16.5	67 9 172
drmarthagulati	599	114	6.5	23.0	25 639
jaccjournals	224	36	2.4	25.4	36 497
accmediacenter	157	90	1.7	27.2	12 385
circaha	151	7	1.6	28.8	37 533
cardioinfo_it	150	69	1.6	30.4	2285
ahascience	146	35	1.6	32.0	56 207
cardiologytoday	144	66	1.6	33.6	40 150
pooh_velagapudi	111	12	1.2	34.8	7391
nadeenfaza	104	4	1.1	35.9	4066
hadleywilsonmd	103	22	1.1	37.0	595
dfcapodanno	103	10	1.1	38.2	3178
gilberttangmd	99	10	1.1	39.2	2056
mirvatalasnag	97	16	1.1	40.3	8681
dlbhattmd	95	37	1.0	41.3	12 848
sabouretcardio	94	8	1.0	42.4	5054
aayshacader	91	12	1.0	43.3	2006
vallealfonso	87	8	0.9	44.3	6814
fischman_david	84	24	0.9	45.2	13 082

*Note:* Source: NodeXL.

**FIGURE 2:**
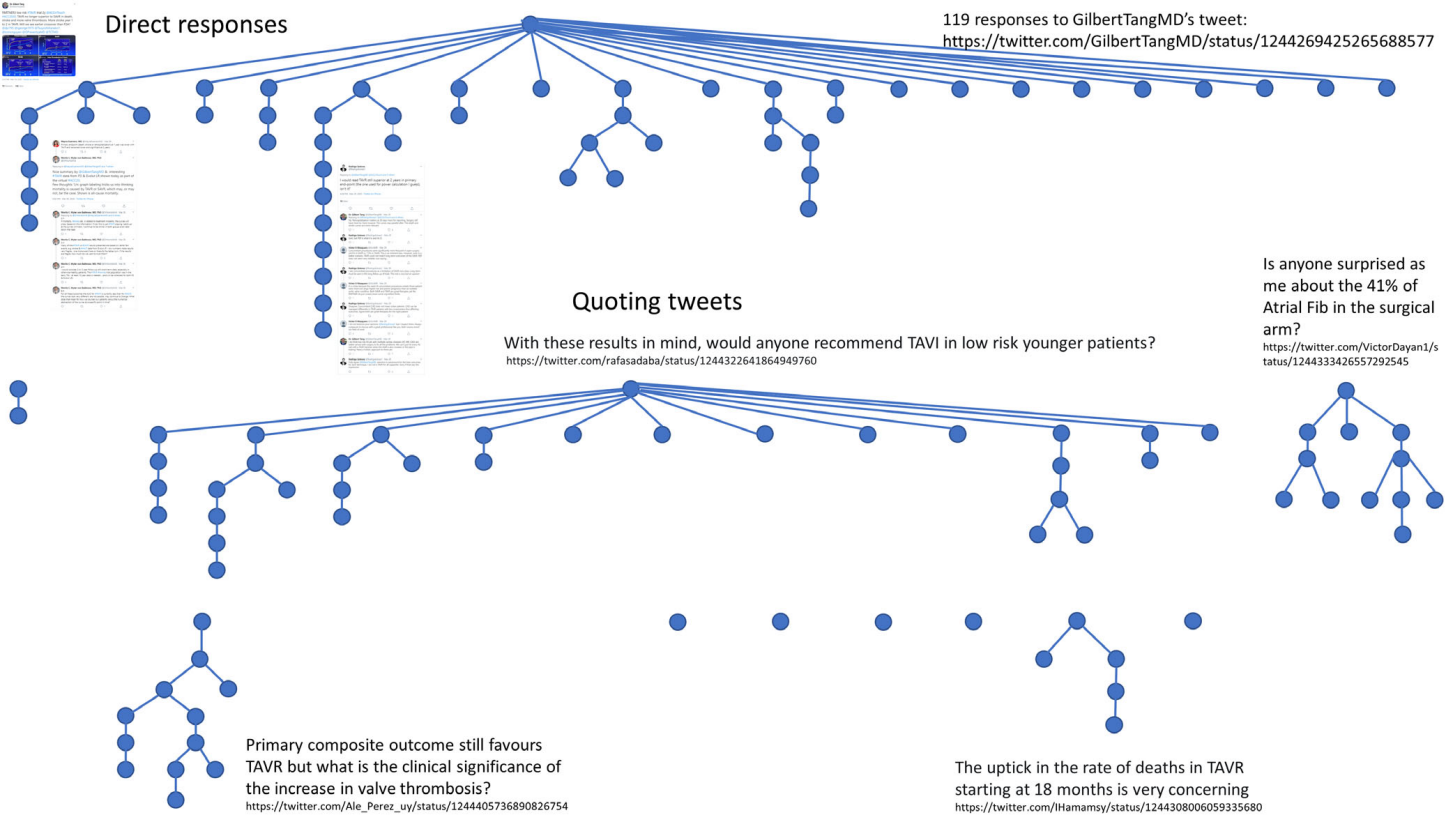
Responses to GilbertTangMD's tweet^5^(Source: Manual searching of Twitter.com)

Dr Gilbert Tang's tweet on the PARTNER 3 study comparing transcatheter aortic valve replacement (TAVR) and surgical aortic valve replacement (SAVR) received 119 responses (Figure 2), including 52 replies to the original tweet, 10 tweets quoting Dr Tang's original tweet,^5^ and 57 subsequent replies to these quoting tweets. These responses were posted by Dr Tang and 54 others, 46 of whom did not post other tweets related to ACC.20/WCC. The number of responses exceeded the number of retweets received by Dr Tang's original tweet (n = 70 retweets). Many of the responding tweets unpicked the details of the selected study (PARTNER3), performing the function of a critical appraisal, sometimes with an individual posting a thread of tweets explaining a point in detail, and other times in a dialog between two or more tweeters. Topics discussed included the age and comorbidities of study participants, selected outcome measures, and longer‐term results. Of note, the original tweet used the #ACC2020 hashtag rather than the official #ACC20 hashtag. Of the 119 responses, only 4 included #ACC2020 and none included the #ACC20 hashtag. Accordingly, 5 of the 119 responses were not included in the original NodeXL extract searching for ACC.20/WCC related hashtags. The 5 responses that included the #ACC2020 hashtag received 27 retweets, while the remaining 114 responses received a total of 56 retweets.

Symplur data (Figure S2) provide data on tweets and tweeters using the #ACC20 hashtag, which in fact combine data on retweets and retweeters, respectively. These figures are higher than data recorded by NodeXL, even though the NodeXL extract included additional hashtags. The number of impressions estimated by Symplur is likely to be a large over‐estimate. Twitter Analytics records impressions for tweets but not retweets. For the 183 tweets it identified from the @ACCintouch account during the 5 days studied, it records 992  233 impressions. NodeXL recorded 184 tweets by @ACCinTouch over the same period. The @ACCinTouch Twitter feed displays 69 retweets over the same period. This gives a total of 252 tweets and retweets identified directly from Twitter sources rather than third party software. Symplur adds together tweets and retweets to estimate “tweets” and then multiplies this by number of followers to estimate impressions. It records 280 tweets + retweets, 27 or 28 more than the figures direct from Twitter. It is not possible to compare the total estimate of impressions for tweets and retweets estimated by Symplur for @ACCinTouch (Figure S2) with the Twitter Analytics figures. However, for tweets alone, Symplur would estimate 14.8 “impressions” for an account with ACCinTouch's number of followers during the conference period, that is, a 15‐fold overestimate compared with the equivalent figure direct from Twitter Analytics.

## 4 DISCUSSION

This analysis identified reduced Twitter engagement during ACC.20 compared to ACC.19. This finding may be explained by a number of factors including increased clinical workload demands relating to COVID‐19 reducing the time available for education, and alternative forms of virtual engagement (eg, the chat function built into the ACC.20 website) displacing social media activity. Additionally ACC.20 was live for 4 hours a day, for the 3 days, with fewer presentations and posters than in previous years as a result of the change in “venue.” There were no social events tweeted and the number of “selfies” was limited, aside from some postings to share how participants attended ACC.20. As for ACC.19 there was evidence of “hashtag drift,” with a greater proportion of tweeters using the incorrect hashtag (#ACC2020) in 2020 than 2019. Each of these factors reduce opportunities for effective dissemination of scientific findings from a flagship medical event. In an era when “fake news” can trump evidence‐based approaches it is important to maximize sharing and discussion of research and progress in the field of cardiology. There was no “hashtag confusion” evident, with no other events using the #ACC20 hashtag, but this reflects the impact of the COVID‐19 pandemic rather than an active change in hashtag use by other events. In future years the hashtag should be adapted to include a specific cardiology term. One option would be #ACCardio21, ideally matching the name of the conference to the hashtag.

The mapping of responses to Dr Tang's tweet illustrates the very considerable level of interest and dialog that can emerge from a single tweet about new cardiology research findings. The great majority of the participants in this dialog did not tweet otherwise about ACC.20, suggesting dissemination well beyond the virtual event, with the topic matter and connections within an existing social network becoming as important as the originating event. Social media provides a mechanism for clinicians and researchers to reach a wide audience, with opportunities for peer review, health promotion and awareness of clinical and scientific advances. Social media can also, however, lead to unwanted attention and offensive responses, sometimes in an orchestrated campaign. The chat function in webinars, including ACC.20, provides a safe environment among peers, but typically lacks a permanent record or the social networking features so effectively implemented by Twitter and other platforms. There is a balance to be achieved between these different approaches.

There are strengths to this analysis. The ability to look beyond a single hashtag provides a fuller understanding of the social media activity around the virtual event. The separation of tweeting from retweeting allowed by extracting raw data provides a better understanding of the split between content generation (tweeting) and dissemination (retweeting). The Wakelet summaries produced as a by‐product of both ACC.19 and ACC.20 provide a visual summary of the most shared content from the two events that can be used for continuing professional development with a much wider audience. The detailed description of responses to Dr Gilbert Tang's tweet demonstrates the importance of looking beyond the hashtag to understand the wider dialog emerging around new research. There are also limitations, including the shortfall in number of individual retweets recorded by NodeXL, though importantly this has not been observed to impact on number of tweets recorded.^11^ Symplur healthcare hashtags is used commonly during conferences to produce a snapshot of Twitter activity, while the more detailed analysis using NodeXL takes a few hours to complete, including data extraction and analysis time. As with any research activity, knowing operational definitions and being able to reproduce findings is important.

The data obtained for this analysis of ACC.20 via NodeXL identifies clearly the breakdown of tweets and retweets and demonstrates the great over‐estimation of Twitter “impressions” by Symplur, and a smaller amplification of number of tweets and retweets. These lessons have applications to analysis of social media activity at future cardiology educational events following the COVID‐19 pandemic. The global cardiology community continued to communicate despite COVID‐19, with the virtual event complemented by tweeting and other technology.

## Supporting information


**Figure S1**. Number of retweets made, comparing equivalent 5‐day period for ACC.19 and ACC.20 (Source: NodeXL).

**Figure S2**. Symplur data for #ACC20 hashtag (Source: Symplur healthcare hashtags website^8^).

**Data S1**. NodeXL records information on retweets in two ways ‐ both as a total number of retweets for individual retweets, and as records of individual retweets. The NodeXL extract used in this analysis was recorded a few hours after the period of study: the aggregate estimate of number of retweets recorded in individual tweets was 9189 retweets, while the number of individual retweets recorded was 8566 retweets, representing ~93% of the expected total. The shortfall is not explained by retweeting in the period from midnight on April 1, 2020 to the time of extract, as only 74 retweets of tweets posted during the preceding 5 days were recorded over this 6‐hour period.
