# Corrigendum

**DOI:** 10.1002/clc.23491

**Published:** 2020-10-29

Mackenzie G, Gulati M. ACC.20: Impact of social media at the virtual scientific sessions during the COVID‐19 pandemic. Clin Cardiol. 2020 Sep;43(9):944‐948. doi: 10.1002/clc.23387.

For the article cited above, there is an error in the sentence:

“However, for tweets alone, Symplur would estimate 14.8 ‘impressions’ for an account with ACCinTouch's number of followers during the conference period, that is, a 15‐fold overestimate compared with the equivalent figure direct from Twitter Analytics.”

Should have read as:

“However, for tweets alone, Symplur would estimate 14.8 million ‘impressions’ for an account with ACCinTouch's number of followers during the conference period, that is, a 15‐fold overestimate compared with the equivalent figure direct from Twitter Analytics.”

We apologize for this error.

