# Supplementary figures and images for: ACC.20: Impact of social media at the virtual scientific sessions during the COVID‐19 pandemic
Source: Clin Cardiol. 2020 Jul 3;43(9):944–8. doi: 10.1002/clc.23387 (PMC7462185; doi:10.1002/clc.23387)

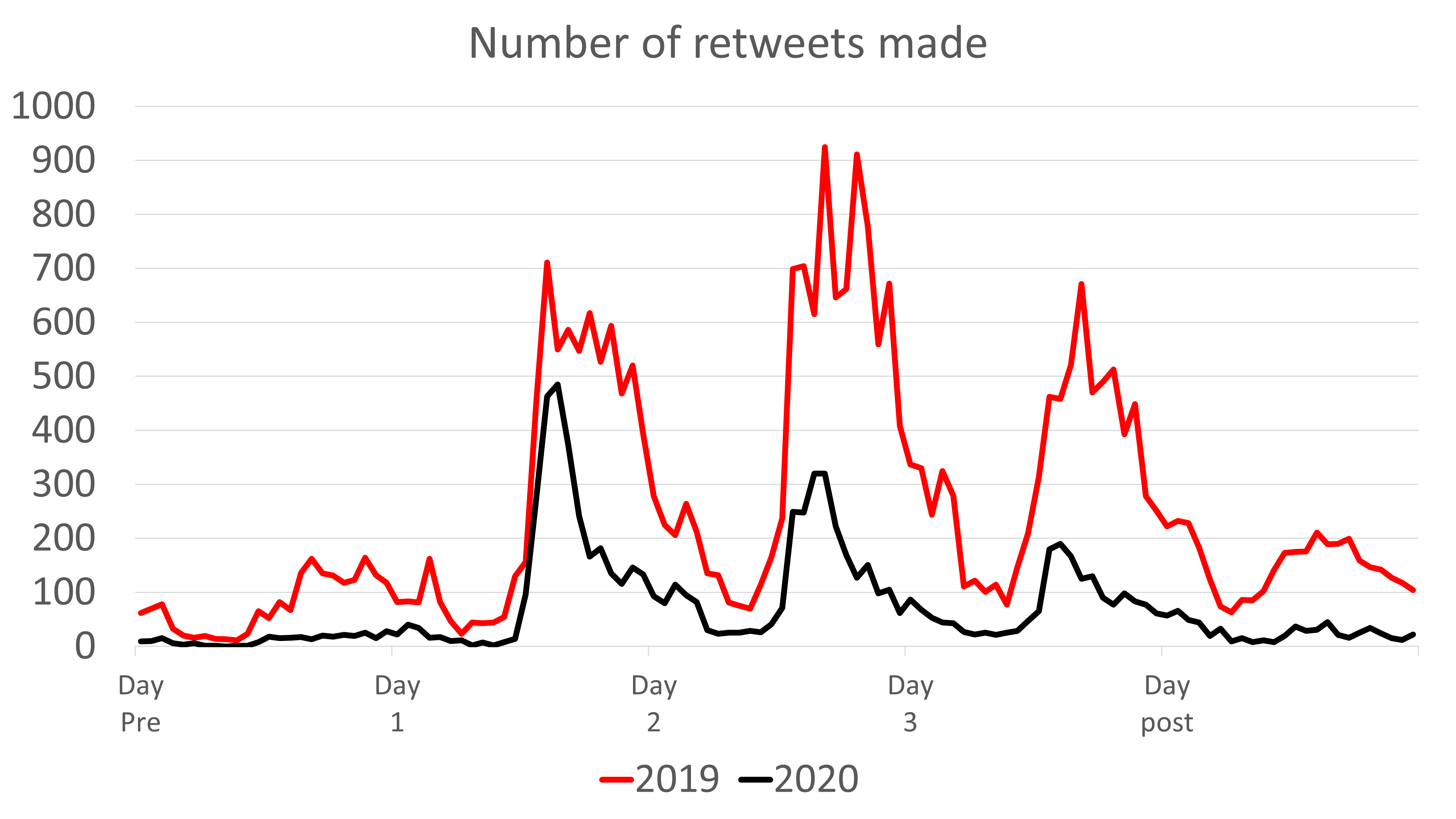

Supplement: Supplementary file 1 — Figure S1. Number of retweets made, comparing equivalent 5‐day period for ACC.19 and ACC.20 (Source: NodeXL). [file CLC-43-944-s001.tif]

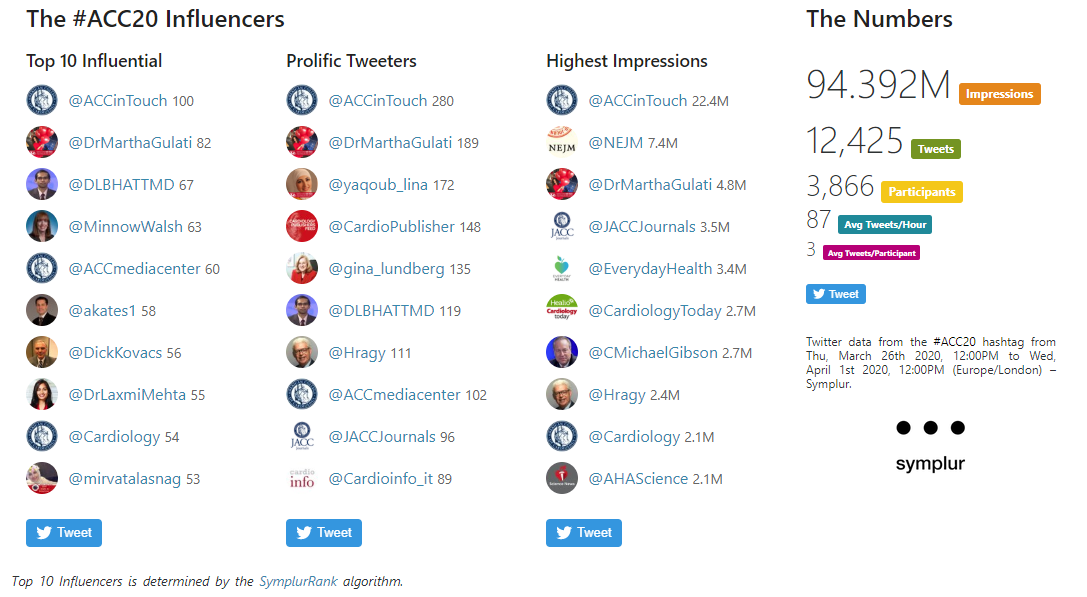

Supplement: Supplementary file 2 — Figure S2. Symplur data for #ACC20 hashtag (Source: Symplur healthcare hashtags website 8 ). [file CLC-43-944-s002.png]
