# Supplementary material for: ACC.20: Impact of social media at the virtual scientific sessions during the COVID‐19 pandemic
Source: Clin Cardiol. 2020 Jul 3;43(9):944–8. doi: 10.1002/clc.23387 (PMC7462185; doi:10.1002/clc.23387)
Supplement: Supplementary file 3 — Data S1. NodeXL records information on retweets in two ways ‐ both as a total number of retweets for individual retweets, and as records of individual retweets. The NodeXL extract used in this analysis was recorded a few hours after the period of study: the aggregate estimate of number of retweets recorded in individual tweets was 9189 retweets, while the number of individual retweets recorded was 8566 retweets, representing ~93% of the expected total. The shortfall is not explained by retweeting in the period from midnight on April 1, 2020 to the time of extract, as only 74 retweets of tweets posted during the preceding 5 days were recorded over this 6‐hour period. [file CLC-43-944-s003.docx]

Data S1. NodeXL records information on retweets in two ways - both as a total number of retweets for individual retweets, and as records of individual retweets. The NodeXL extract used in this analysis was recorded a few hours after the period of study: the aggregate estimate of number of retweets recorded in individual tweets was 9189 retweets, while the number of individual retweets recorded was 8566 retweets, representing ~93% of the expected total. The shortfall is not explained by retweeting in the period from midnight on April 1, 2020 to the time of extract, as only 74 retweets of tweets posted during the preceding 5 days were recorded over this 6-hour period.
